# Supplementary material for: Structure-function analysis of the cyclic β-1,2-glucan synthase from Agrobacterium tumefaciens
Source: Nat Commun. 2024 Feb 28;15:1844. doi: 10.1038/s41467-024-45415-8 (PMC10901819; doi:10.1038/s41467-024-45415-8)
Supplement: Supplementary file 1 — Supplementary Information [file 41467_2024_45415_MOESM1_ESM.pdf]

Supplementary Information

Structure-function analysis of the cyclic β-1,2-glucan synthase from

Agrobacterium tumefaciens

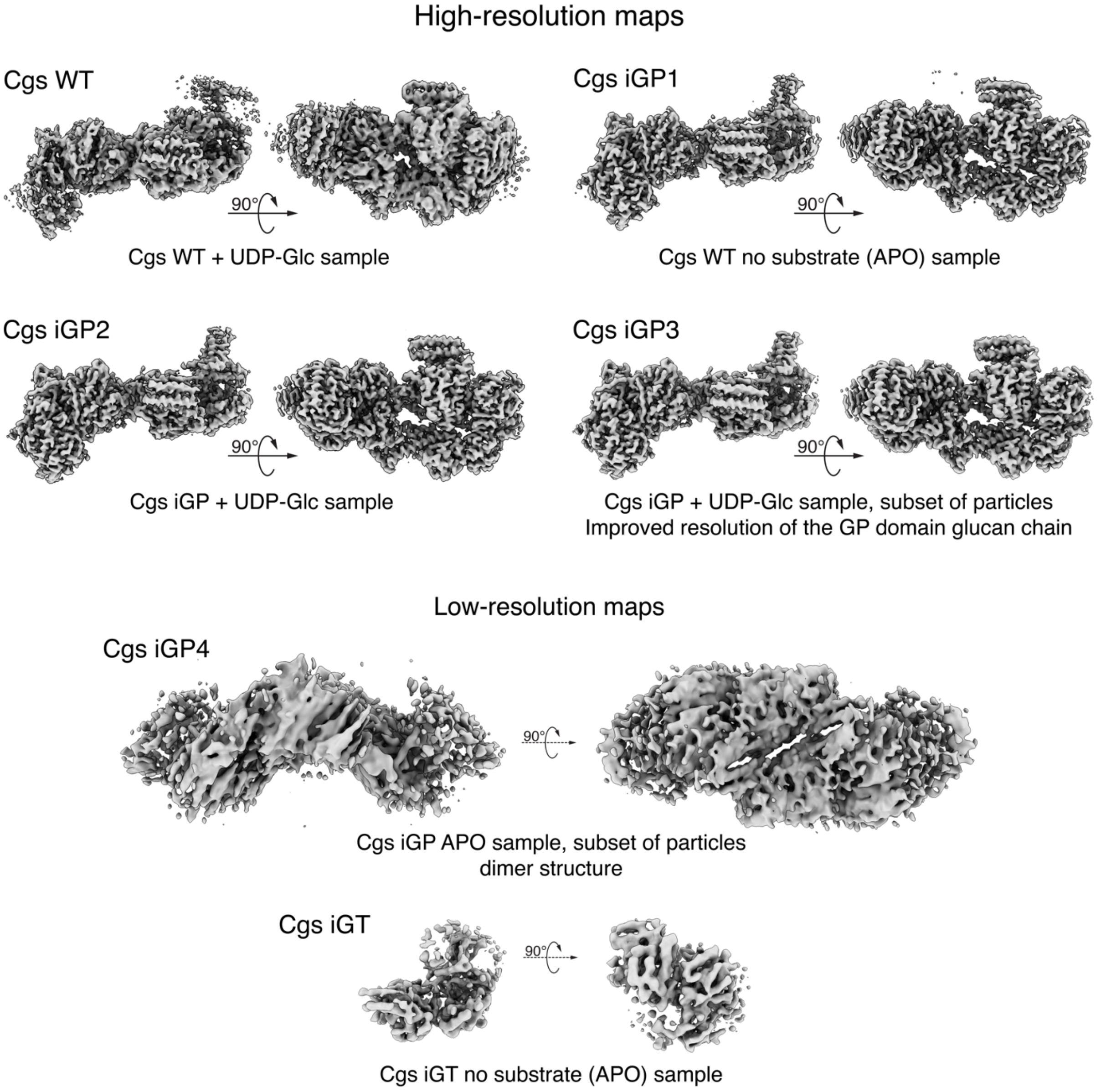

Supplementary Fig. 1 | Cryo-EM density maps obtained in this study. The maps were generated using the wild-type (Cgs_{WT}), inactive GP (Cgs_{iGP1}, Cgs_{iGP2}, Cgs_{iGP3}, Cgs_{iGP4}) and inactive GT84 (Cgs_{iGT}) protein samples. Cgs_{WT}, Cgs_{iGP1} and Cgs_{iGP2}, Cgs_{iGP3} are high-resolution maps of the full-length protein. Cgs_{iGP4} represents a homodimer. Cgs_{iGT} is a low-resolution map of the N-terminal half of the protein.

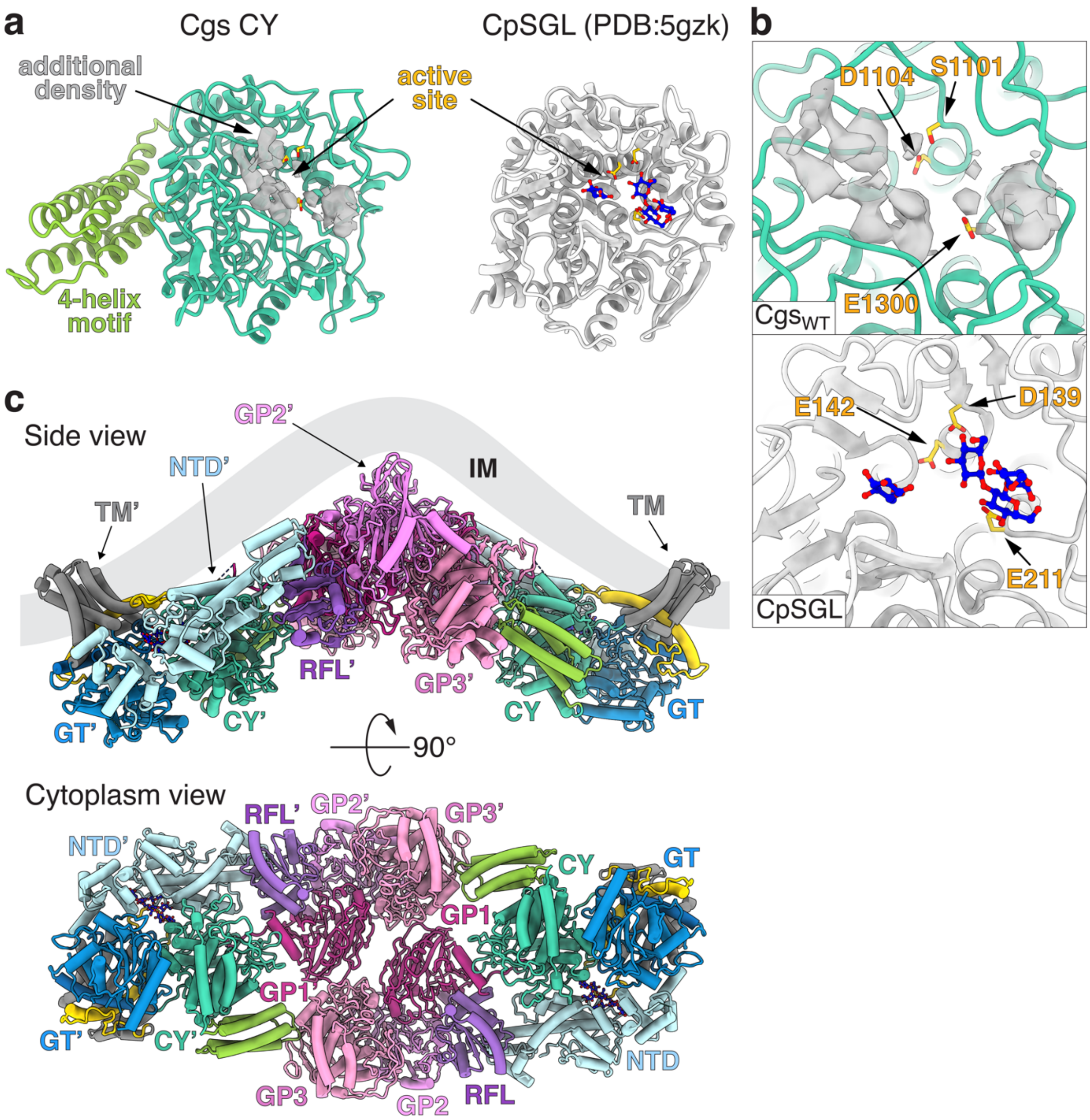

Supplementary Fig. 2 |Characterization of the CY domain and its interactions. a, Comparison of the CY domain of Cgs (left) and the soluble GH144 family homolog CpSGL (5GZK). Residues relevant for the function were marked in yellow. An additional density (gray), likely corresponding to a fragment of a glucan chain, was found in the active pocket of the CY domain in the Cgs$_{WT}$ map. The sophorotriose and glucose molecules co-crystallized with CpSGL are shown as blue-red sticks. The 120-residue part constituting the 4-helix domain (green) is absent in the homolog. **b,** Close up on the enzymatic pockets of CY and CpSGL. Residues S1101, D1104 and E1300, which correspond to D139, E142 and E211 of CpSGL, were shown to play a role in Cgs activity. **c,** Side (top) and cytoplasm (bottom) views of the Cgs dimer structure. Cgs forms a back-to-back dimer through the interaction of the 4-helix motif with the back of the GP. The angle between the TMs of both monomers indicates that the accommodation of the dimer in the inner membrane (IM) could be potentially associated with increased curvature.

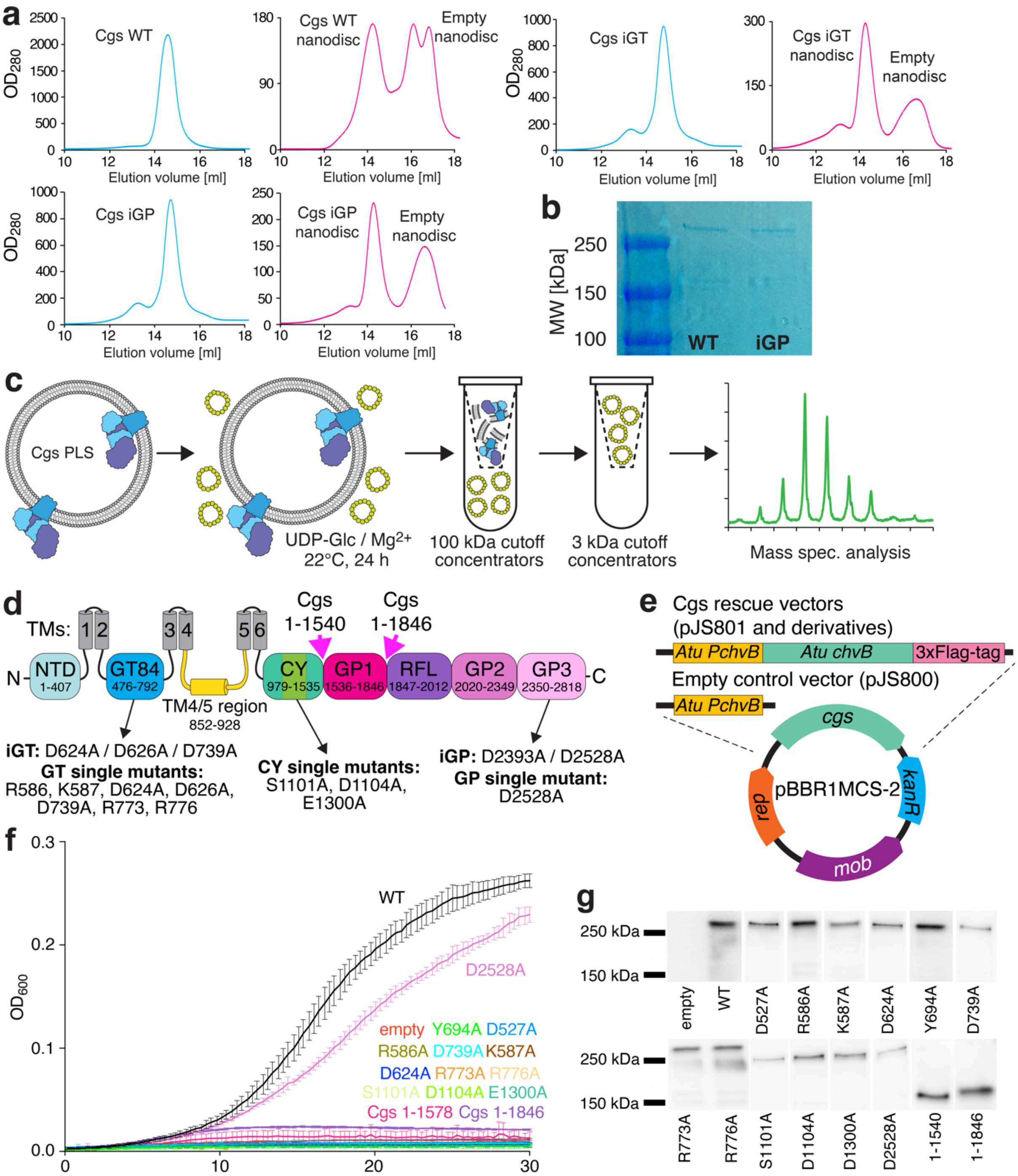

Supplementary Fig. 3 | Cgs activity. a, SEC profiles of Cgs in DDM micelles (blue) and MSP1D1 nanodiscs (magenta). (top-left) Cgs_{WT}; (top-right) Cgs_{iGT}; (bottom) Cgs_{iGP}. **b,** SDS-PAGE of Cgs proteoliposomes (PLS) indicating the incorporation of the protein (320 kDa). **c,** Diagram describing the in vitro CβG synthesis assay. **d,** Diagram of Cgs topology indicating the mutants discussed in the study. Location of stop codons that resulted in truncation mutants is indicated (magenta arrows). **e**, Cartoon representation of the constructs for the hypoosmotic stress assay (HOS). **f,** Growth dynamics of *Atu* in the HOS. The *Δcgs* mutant was rescued with different constructs of the *cgs* gene. **g**, Western blot analysis of *cgs-3xFlag* gene expression in *Atu* strains used in the HOS. Error bars represent standard deviation (SD) of three biological replicates (n=3). Data for graph f and uncropped images for g are available as source data. Western blot analysis was performed once (n=1). Source data are provided as a Source Data file.

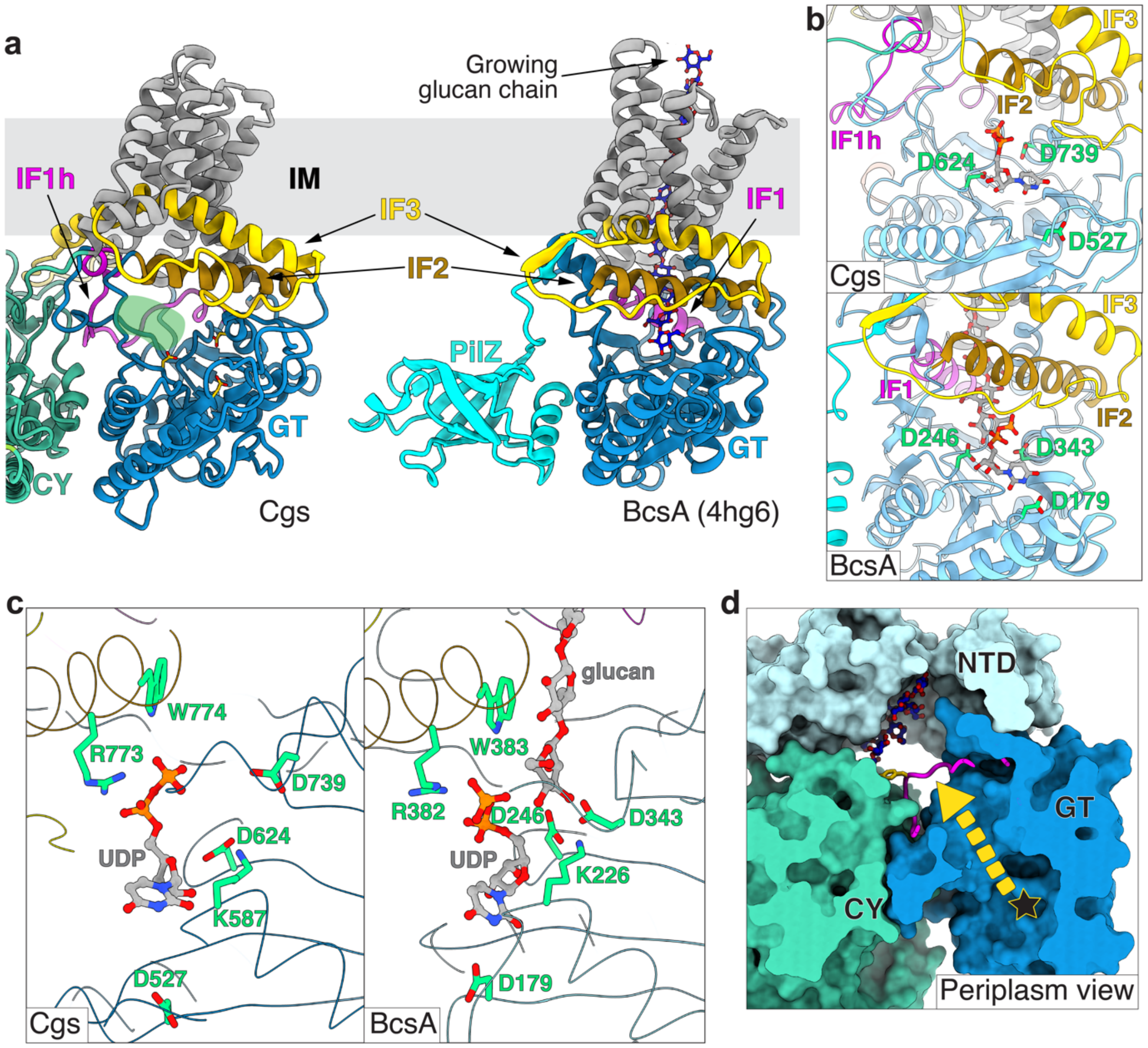

Supplementary Fig. 4 | Comparison of the GT84 domain with GT2 family glycosyltransferases. a, Comparison of Cgs (left) and BcsA (4HG6, right). The PilZ domain (cyan), responsible for cdGMP-dependent regulation of BcsA, is absent in Cgs. The interface motifs IF1 (magenta), IF2 (brown) and IF3 (yellow) are indicated. In BcsA, the synthesized sugar chain is translocated across a channel formed by the TM domain. The compact TM of Cgs does not form a channel. Instead, the unfolded IF1-homology (IF1h) loop opens a channel towards the other side of the GT domain (green shading). **b,** Close-up of the GT active sites of Cgs (left) and BcsA (right). Coloring as in a. Active site residues important for the function of Cgs GT and the respective equivalents in BcsA are shown in green. **c**, Comparison of the enzymatic pocket composition of Cgs GT and BcsA. Residues that are conserved between Cgs and BcsA are shown in green. **d**, Cross-section of the channel leading from the active site of the Cgs GT (star) towards the NTD, shown from the periplasmic side. The arrow indicates the suggested path of the growing glucan chain.

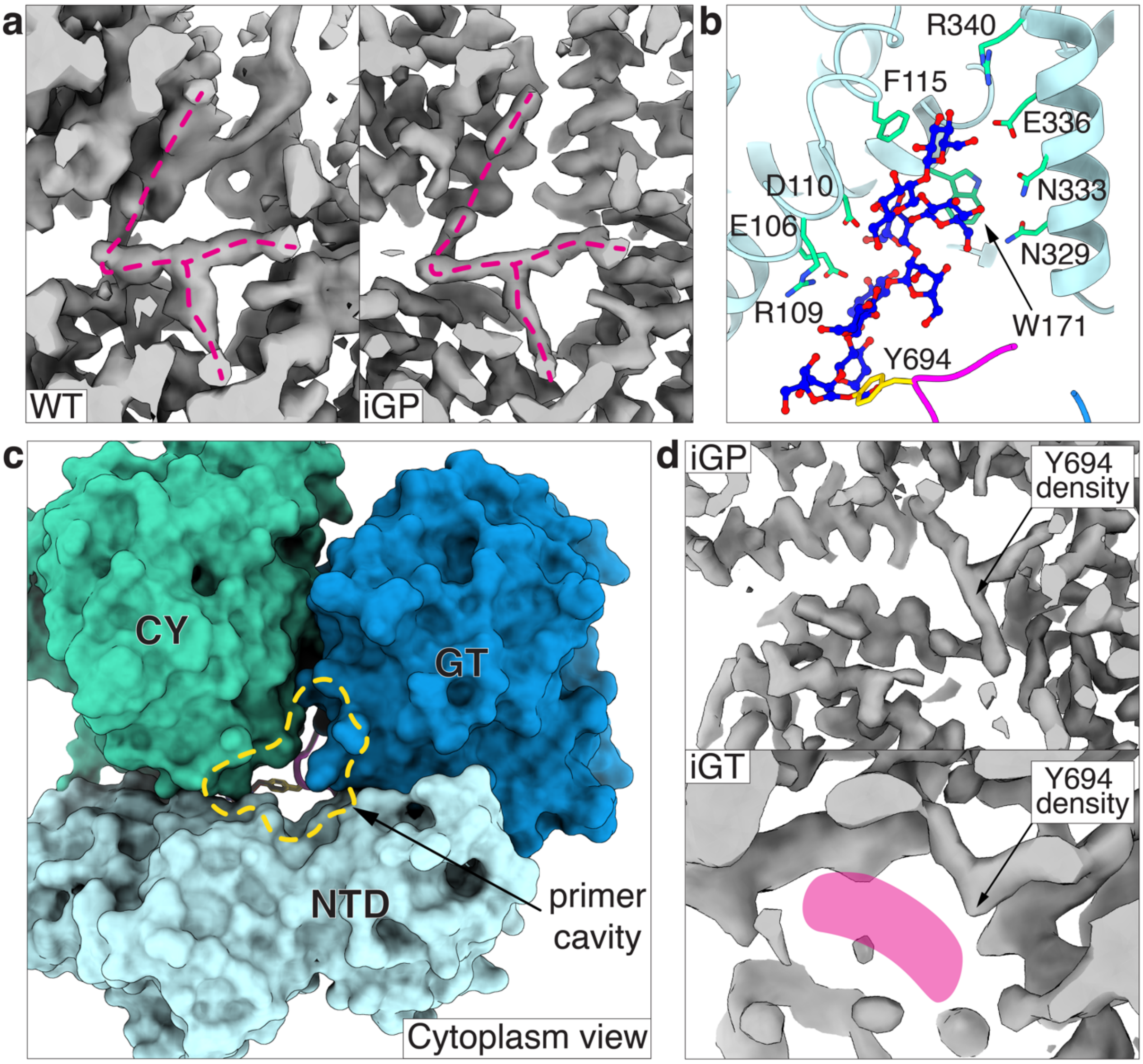

Supplementary Fig. 5 | Autoglycosylation of Cgs. a, Cryo-EM maps obtained for the WT (left) and iGP (right) constructs. The magenta line indicates the Y-junction corresponding to the glycosylated IF1h loop at Y694. **b,** Close-up of the O-glycan (blue-red sticks) coordinated in the NTD TPR-homology fold. Residues interacting with the glucan chain are indicated in cyan. **c,** Close-up of the NTD/GT/CY interface seen from the cytoplasmic side. A large cavity (yellow line) allows access to the primer chamber from the cytoplasm. **d,** Close up of the O-glycosylation site in the iGP (top) and iGT (bottom) maps. In the low-resolution iGT structure, the volume occupied by the glucan chain appears empty (magenta).

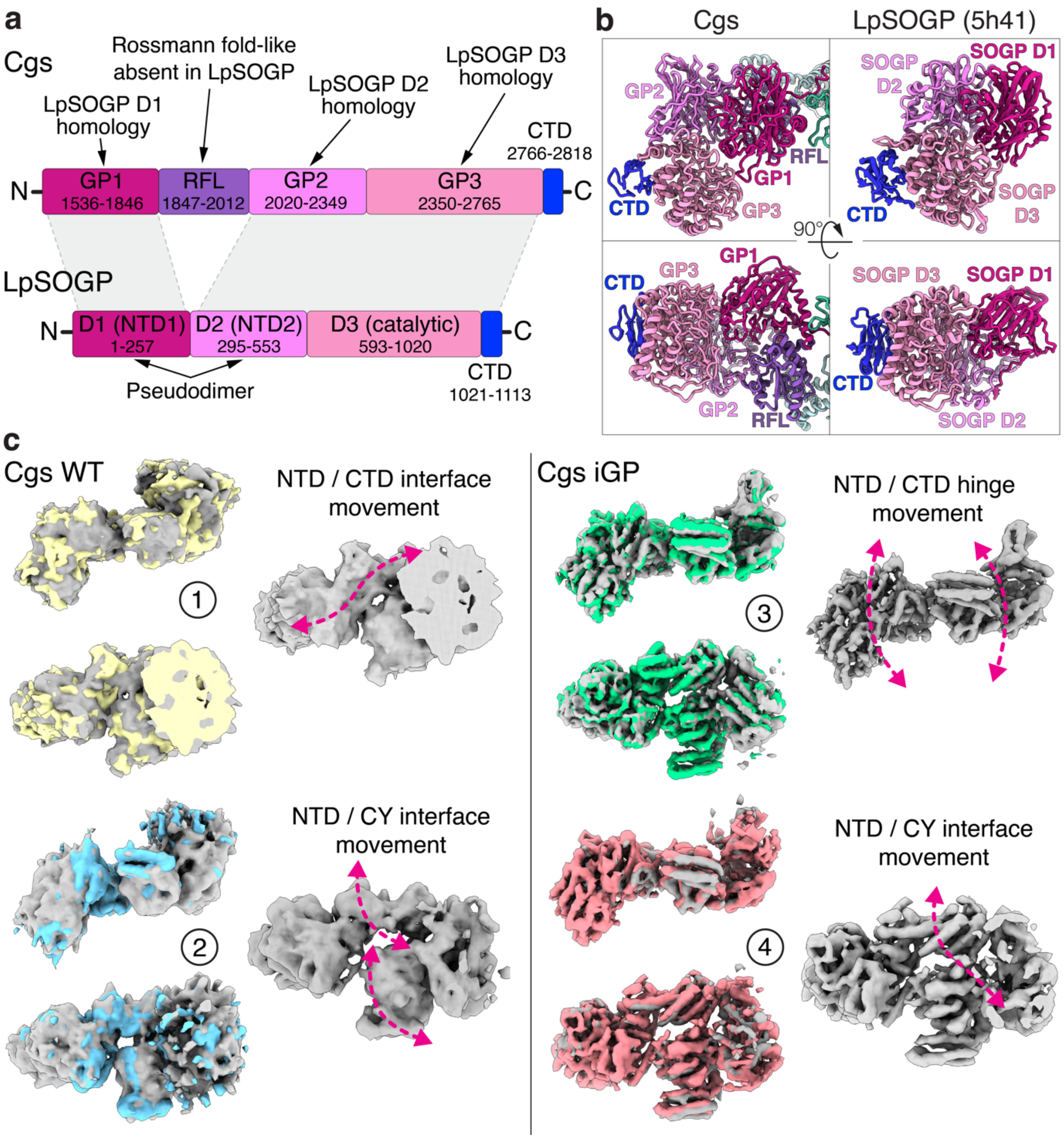

Supplementary Fig. 6 | Cgs CTD. a, Comparison of the domain organization of Cgs CTD (left) with the GH94 family β-1,2-glucan phosphorylase LpSOGP (right). The Cgs CTD can be divided into four subdomains, three of which (GP1, GP2, GP3) are homologous to the subdomains in LpSOGP. An additional Rossmann-like fold subdomain (purple) is inserted between the GP1 and GP2 of Cgs. **b,** Structure comparison of the domains from a. GP1 and GP2 of both proteins are characterized by a pseudodimeric arrangement. **c,** 3D variability analysis of Cgs_{WT} (left) and Cgs_{iGP} (right) maps. Large domain movements are indicated with arrows. Relative domain movement leads to changing distance at the interfaces between NTD and CTD (1), as well as NTD and CY (2, 4). A hinge movement between the N- and C-terminal halves of Cgs can be observed (3). The opening between the NTD and CY in some conformations could allow access from the primer chamber to the CY active site.

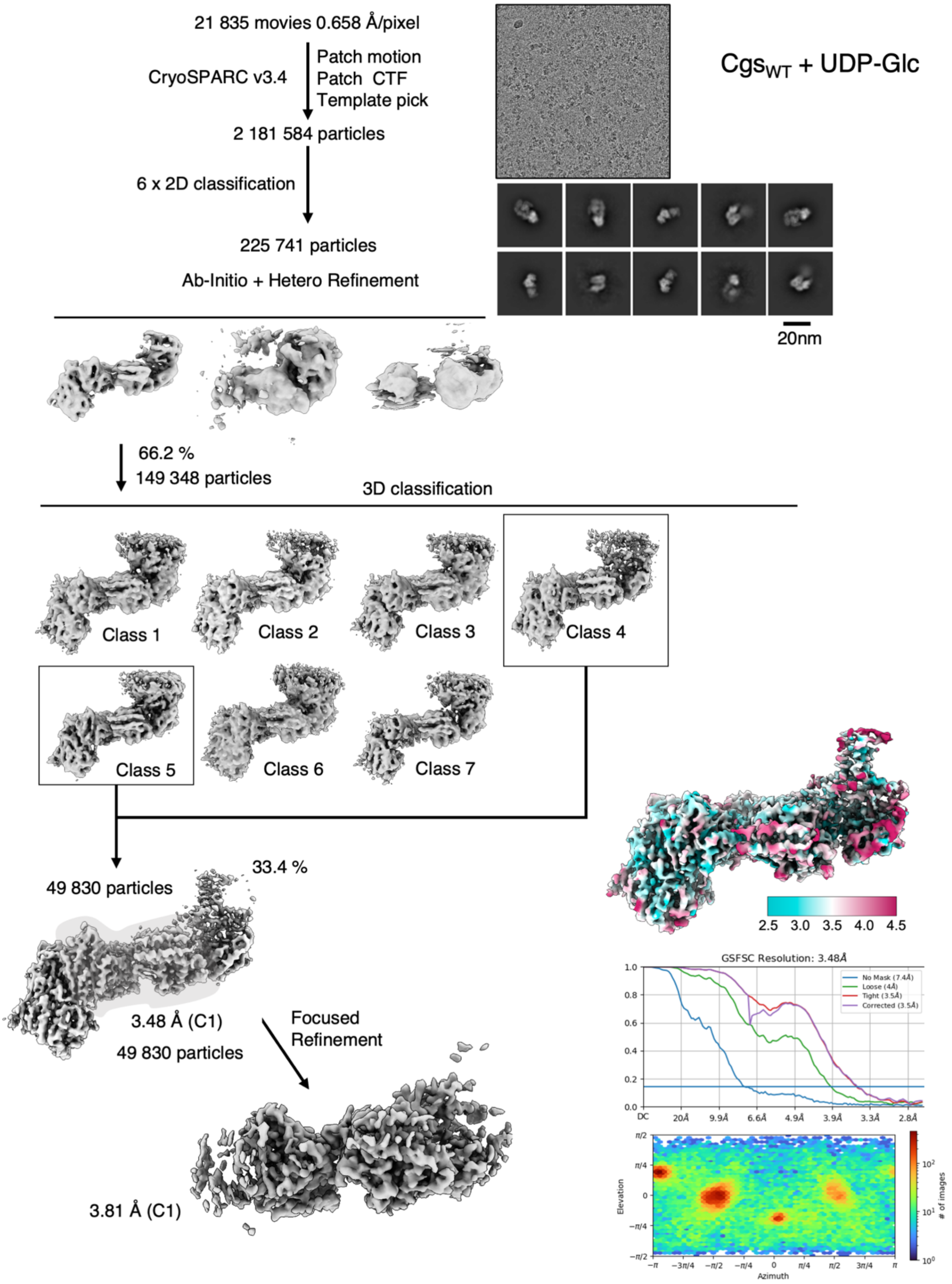

Supplementary Fig. 7 | Single particle processing workflow of the dataset obtained with the Cgs$_{WT}$ UDP-Glc sample

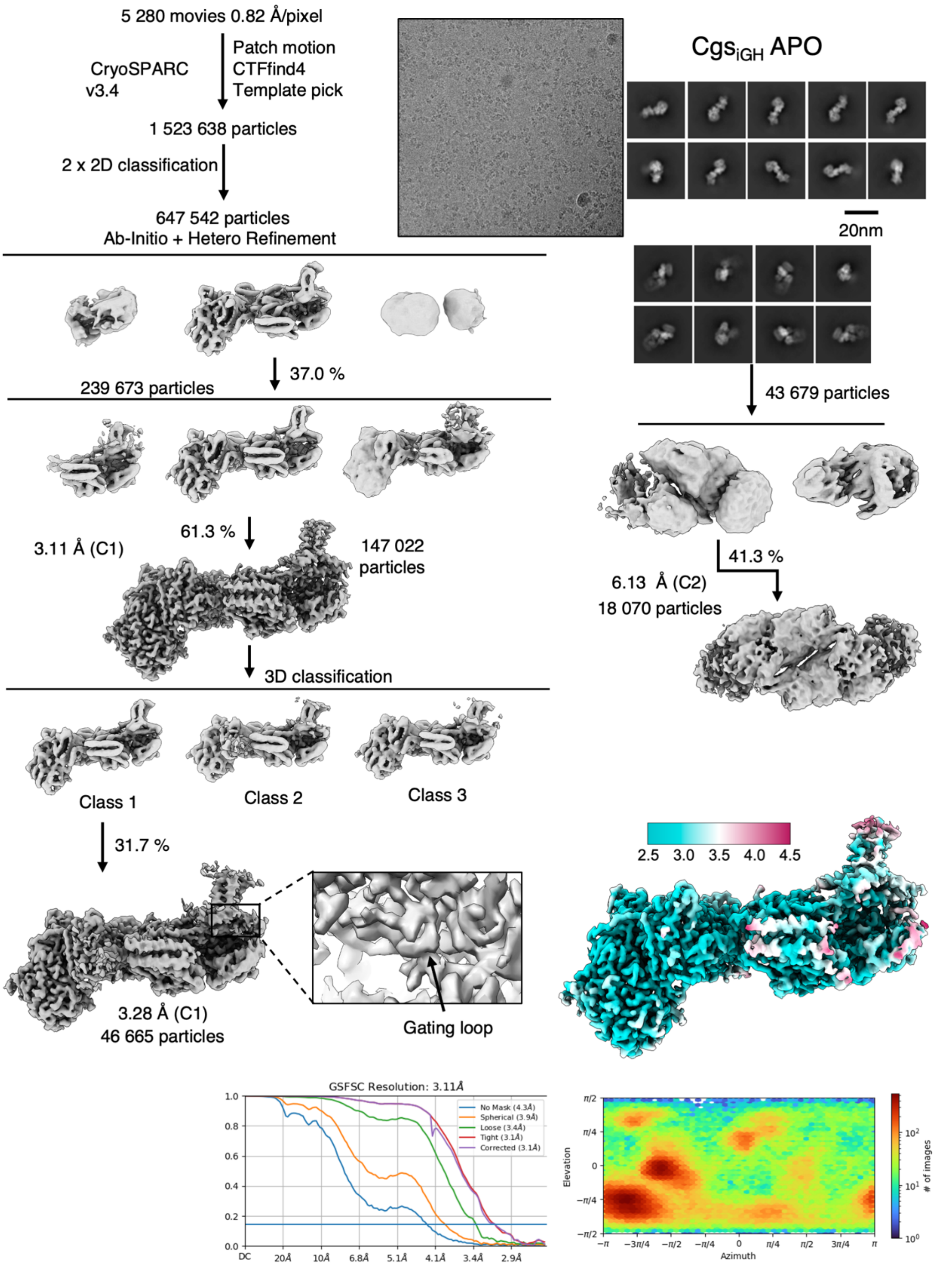

Supplementary Fig. 8 | Single particle processing workflow of the dataset obtained with the Cgs_{iGP} APO sample

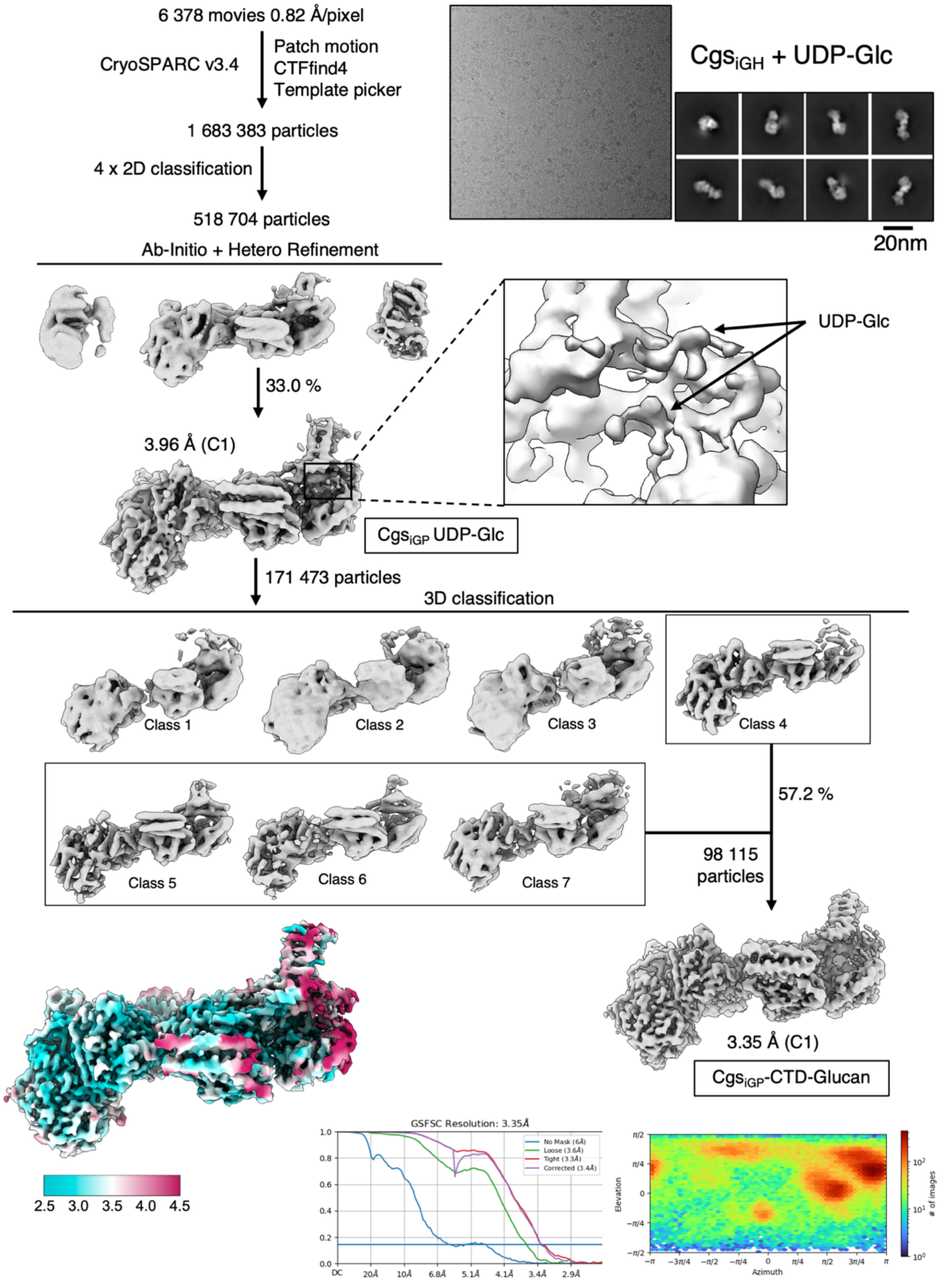

Supplementary Fig. 9 | Single particle processing workflow of the dataset obtained with the Cgs$_{iGP}$ UDP-Glc sample

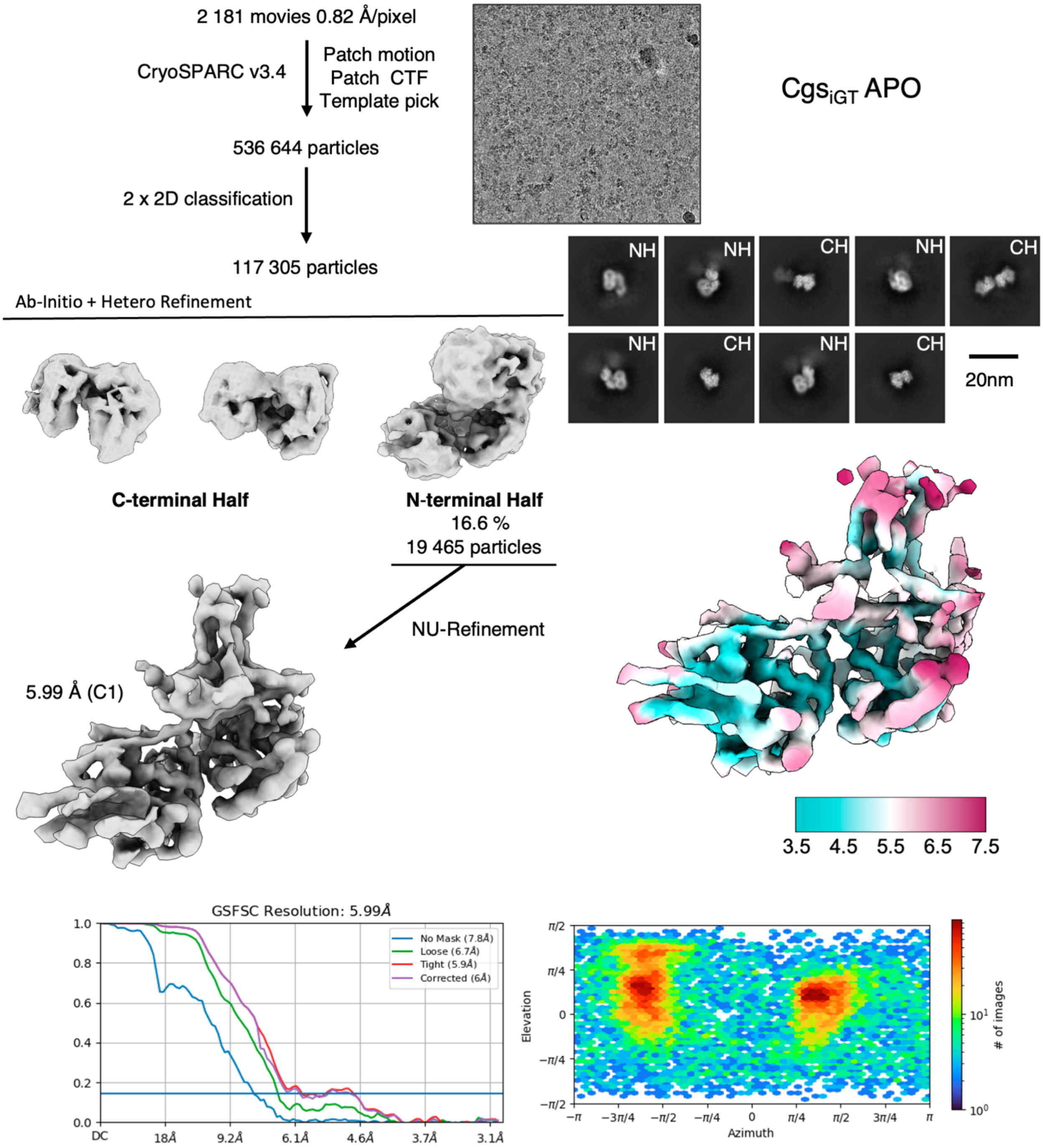

Supplementary Fig. 10 | Single particle processing workflow of the dataset obtained with the Cgs$_{iGT}$ sample

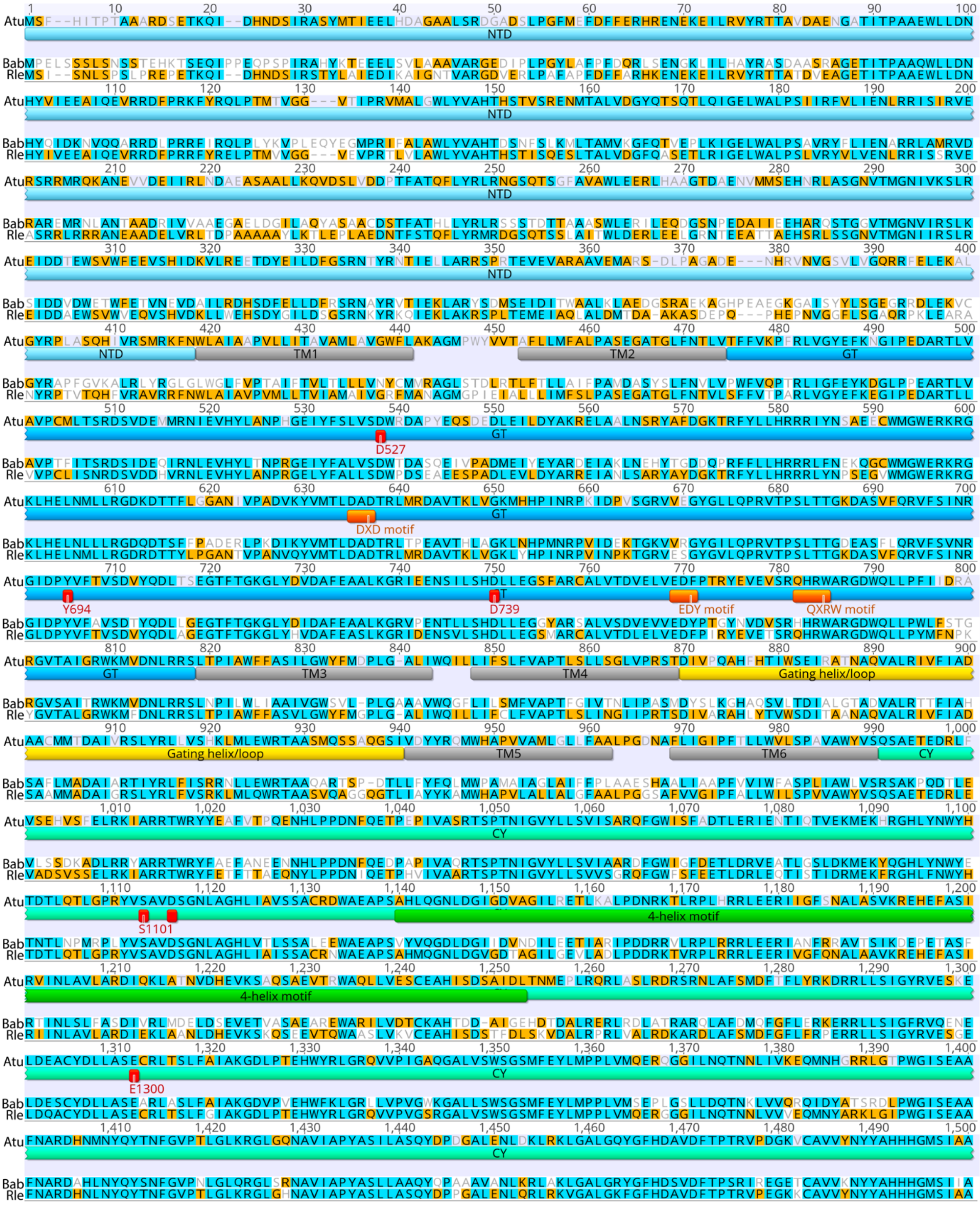

Atu
Bab
Rle
NTD
TM1
TM2
GT
D527
DXD motif
Y694
D739
EDY motif
QXRW motif
TM3
TM4
Gating helix/loop
TM5
TM6
CY
S1101
4-helix motif
E1300

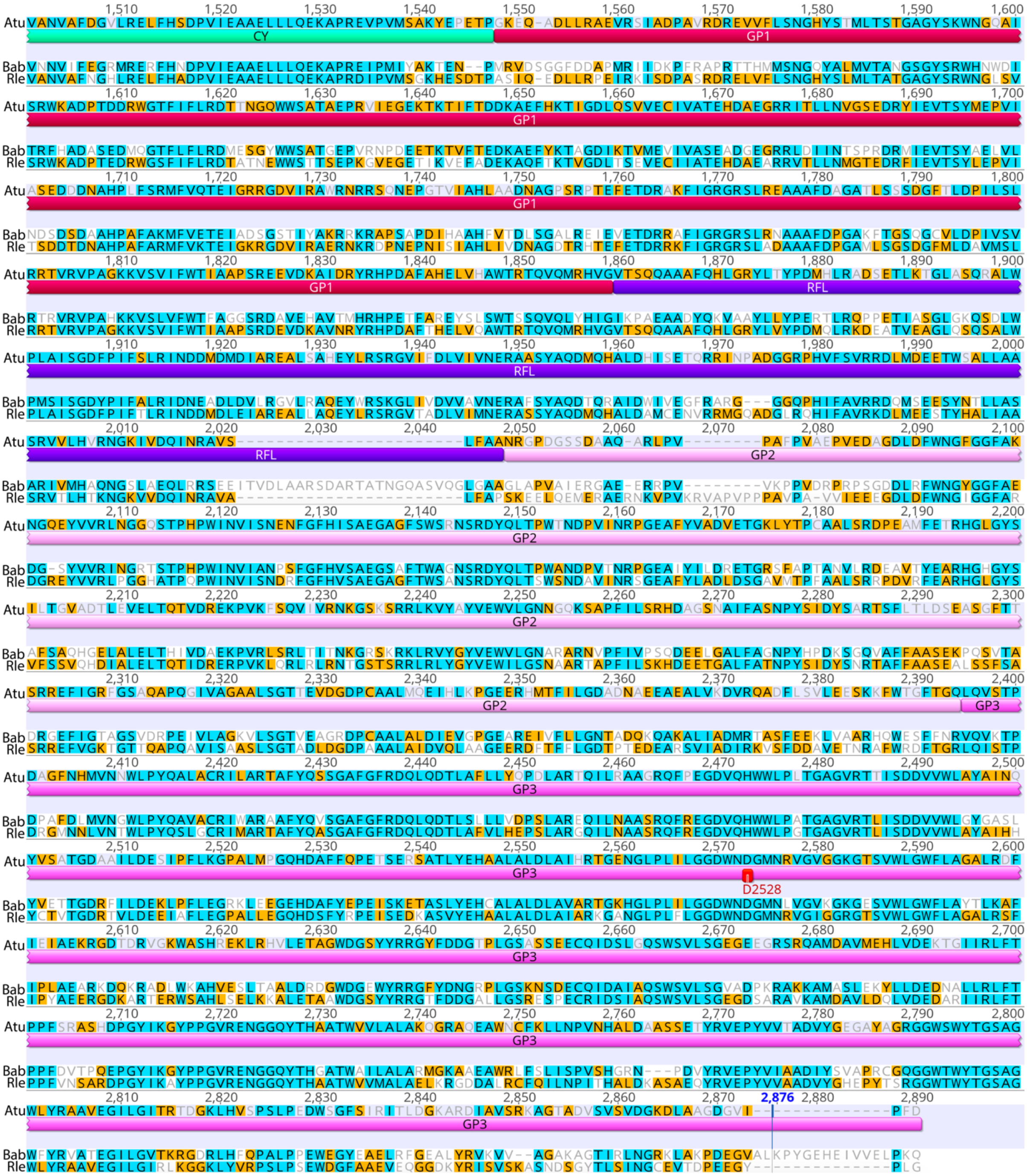

Supplementary Fig. 11 | Sequence alignment of Cgs (NdvB) from *Agrobacterium tumefaciens* (*Atu*), *Brucella abortus* (*Bab*) and *Rhizobium leguminosarum* (*Rle*), indicating the conservation of the protein across *Rhizobiales*. Domain annotation coloring follows the scheme of Fig. 1. Key residues (red) and motifs (orange) are indicated. Residue numbering taken from *Atu* Cgs. Alignments were generated using Geneious Prime 2023.

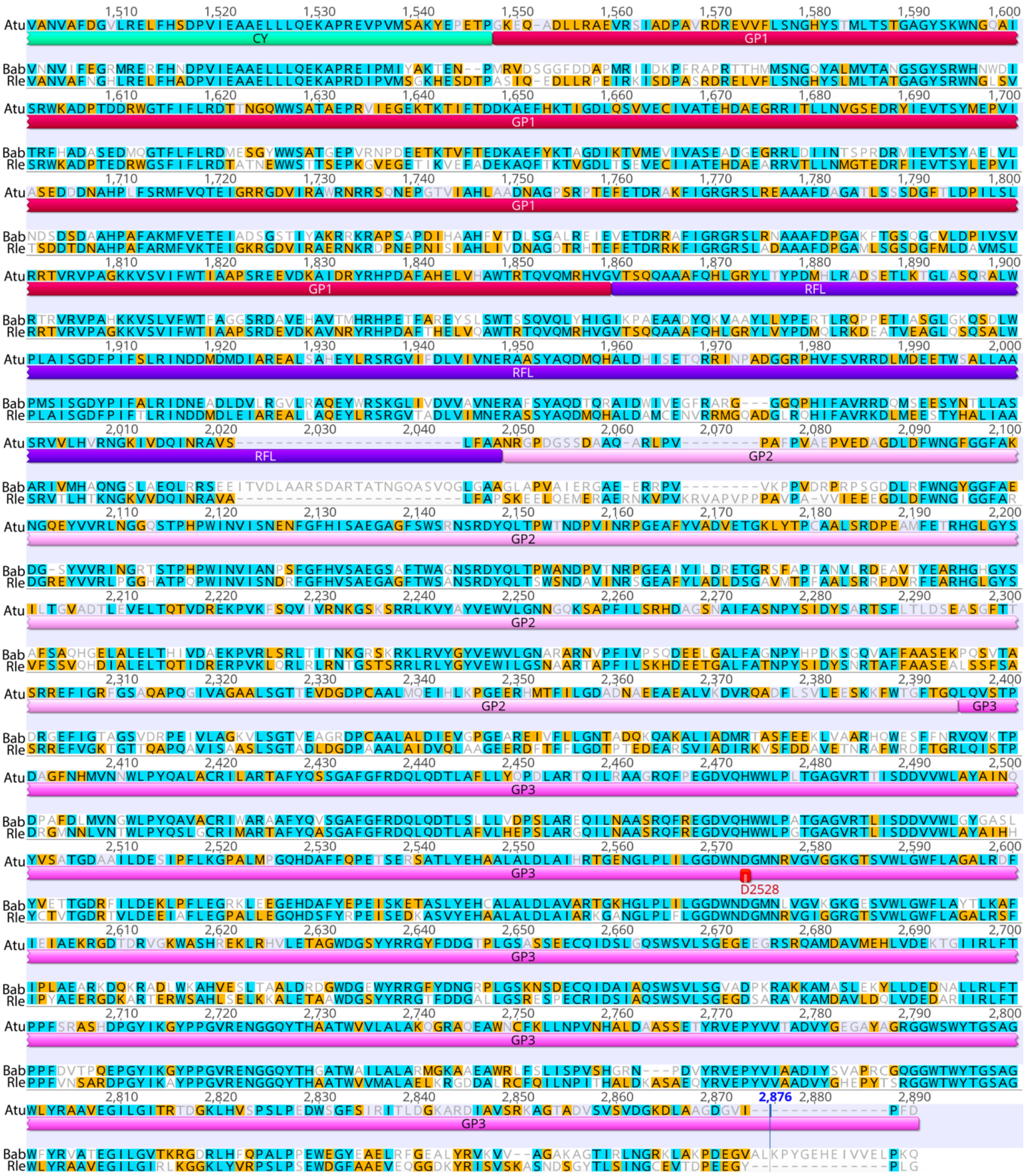

Supplementary Fig. 12 | Sequence alignment of the Cgs CTD with LpSOGP. Domain annotation coloring follows the scheme of Fig. 1. Residue numbering of both proteins is indicated. Alignments were generated using Geneious Prime 2023.

Supplementary Table 1 | Cryo-EM data collection, refinement, and validation statistics

	Cgs WT<br>EMDB-19114<br>PDB 8RF0	Cgs iGP1<br>EMDB-19116<br>PDB 8RF9	Cgs iGP2<br>EMDB-19118<br>PDB 8RFE	Cgs iGP3<br>EMDB-19119<br>PDB 8RFG
**Data collection and processing**				
Magnification	120kx	165kx	165kx	165kx
Voltage (kV)	300	300	300	300
Electron exposure (e–/Å^2)	50	60	60	60
Defocus range (μm)	0.8-2.5	1.0-2.2	1.0-2.2	1.0-2.2
Pixel size (Å)	0.658	0.82	0.82	0.82
Symmetry imposed	C1	C1	C1	C1
Initial particle images (no.)	2 181 584	1 523 638	1 683 383	1 683 383
Final particle images (no.)	49 830	147 022 / 46 665	171 473	98 115
Map resolution (Å)	3.48/3.81	3.11/3.28	3.96	3.35
FSC threshold	0.143	0.143	0.143	0.143
Map resolution range (Å)	30-2.9	30-2.65	30-2.8	30-2.8
**Refinement**				
Initial model used (PDB code)	n/a	n/a	n/a	n/a
Model resolution (Å)	3.55	3.30	4.00	3.45
FSC threshold	0.143	0.143	0.143	0.143
Model resolution range (Å)	30-3.55	30-3.25	30-4.00	30-3.45
Map sharpening *B* factor (Å^2)	-36.9	-100.7	-80	-100.6
Model composition				
Non-hydrogen atoms	21909	21810	21691	21891
Protein residues	2780	2780	2765	1140
Ligands	BGC: 18<br>UDP:1	BGC: 9	BGC: 9<br>UDP:1<br>UDP-GLC:2	BGC: 27
B factors (Å^2)				
Protein	41.89/221.64/91.88	48.52/202.35/90.09	90.56/292.92/140.03	26.20/195.34/66.39
Ligand	90.98/145.44/115.46	72.43/82.38/76.89	75.64/215.73/135.21	42.88/165.11/101.16
R.m.s. deviations				
Bond lengths (Å)	0.002 (0)	0.003 (0)	0.003 (0)	0.004 (0)
Bond angles (°)	0.598 (19)	0.602 (10)	0.707 (13)	0743 (14)
Validation				
MolProbity score	2.06	2.00	2.12	2.08
Clashscore	10.18	8.76	12.66	10.59
Poor rotamers (%)	0.18	0.14	0.18	0.14
Ramachandran plot				
Favored (%)	90.58	90.75	91.59	90.52
Allowed (%)	9.14	8.91	8.30	8.93
Disallowed (%)	0.28	0.34	0.11	0.55

Supplementary Table 2 | Primers used in this study

Name	Sequence from 5' end	Purpose
prJS656	TGAAGCCGGCTGGCGCCAAGCTTCGTCTCCGCCTTGTGATTG	Amplifying the *Atu* chromosome fragment upstream of *chvB* used to generate pJS301
prJS657	GACAGAGACATGTCGTCCGGGCTCGTATTCTGAACTGTATG	Amplifying the *Atu* chromosome fragment upstream of *chvB* used to generate pJS301
prJS658	GACGACATGTCTCTGTCCGC	Amplifying the *Atu* chromosome fragment downstream of *chvB* used to generate pJS301
prJS659	AAGGCCTTGACTAGAGGGTCGACCTCTCGAATGTCTCGGGCAC	Amplifying the *Atu* chromosome fragment downstream of *chvB* used to generate pJS301 and pJS305
prJS697	TGAAGCCGGCTGGCGCCAATGCGGAAGAGGCTGAGG	Amplifying the *Atu* chromosome fragment upstream of *chvB* stop codon; used to generate pJS305
prJS713	GATGTCATGATCTTTATAATCACCGTCATGGTCTTTGTAGTCCATG<br>TCGAAGGGTATAACCCCATCAC	Amplifying the *Atu* chromosome fragment upstream of *chvB* stop codon; used to generate pJS305
prJS714	TATAAAGATCATGACATCGACTACAAGGACGACGATGACAAGTGAG<br>ACGACATGTCTCTGTCCG	Amplifying the *Atu* chromosome fragment downstream of *chvB* stop codon; used to generate pJS305
prJS751	AATAGGCCGACTGCGATG	Amplification of pBBR backbone
prJS926	GCAACTGAGCACGGAAGCC	Amplifying the *Atu* chromosome fragment downstream of D2393 and D2528; used to generate pJS309
prJS927	GGCTTCCGTGCTCAGTTGC	Amplifying the *Atu* chromosome fragment between D2393 and D2528; used to generate pJS309
prJS928	GTTCATGCCGGCGTTCCAG	Amplifying the *Atu* chromosome fragment between D2393 and D2528; used to generate pJS309
prJS929	CTGGAACGCCGGCATGAAC	Amplifying the *Atu* chromosome fragment downstream of D2393 and D2528; used to generate pJS309
prJS1072	ATAAAGATCATGACATCGACTACAAGGACGACGATGACAAGTAGTT<br>CTGCCGACATGGAAGCCATC	Amplification of pBBR backbone
prJS1108	CATCGCAGTCGGCCTATTAAAGTGCTCGTCGCCATGGCG	Amplification of the *chvB* gene and upstream promoter region
prJS1189	TTGAAGCCGGCTGGCGCCAAGCTTGAAGCCATGTTCGAAACCCGTC	Amplifying the *Atu* chromosome fragment downstream of D2393 and D2528; used to generate pJS309
prJS1190	AAGGCCTTGACTAGAGGGTCGACCGAAACATGAAGCTTGCCGTCG	Amplifying the *Atu* chromosome fragment upstream of D2393 and D2528; used to generate pJS309
prJS1272	TCAGGCGGCATGCGGAGGCC	Introducing E1300A mutation to *chvB* gene
prJS1273	CTGGCCTCCGCATGCCGCCTGACC	Introducing E1300A mutation to *chvB* gene
prJS1295	ACAATCGCACACGTCATCATGCAGG	Introducing D895C mutation to *chvB* gene
prJS1296	GATGACGTGTGCGATTGTGCGTTCG	Introducing D895C mutation to *chvB* gene
prJS1297	TGCATGCTGCAGGCCGTGCGCC	Introducing S919C mutation to *chvB* gene
prJS1298	CACGGCCTGCAGCATGCAGTCC	Introducing S919C mutation to *chvB* gene
prJS1306	GTGAAGACAGCAGGATCGATACCGC	Introducing Y694A mutation to *chvB* gene
prJS1307	CGATCCTGCTGTCTTCACCGTTTCC	Introducing Y694A mutation to *chvB* gene
prJS1314	CGCGTAGCGGCAGCAAGGGTCATGAC	Introducing *chvB* D624A / D626A point mutations
prJS1315	CCCTTGCTGCCGCTACGCGCCTGATGC	Introducing *chvB* D624A / D626A point mutations
prJS1316	TCGAGCAGAGCGTGGCTGAGGATGGAG	Introducing *chvB* D739A point mutation
prJS1317	CAGCCACGCTCTGCTCGAAGGCTCC	Introducing *chvB* D739A point mutation
prJS1318	AAGGCCTTGACTAGAGGGTCGACGGTTCCATATTGGTGAGGTCG	Amplifying the *Atu* chromosome fragment used to generate pJS091
prJS1319	TGAAGCCGGCTGGCGCCAAGCTTATGCCGCCGGCACCGATGC	Amplifying the *Atu* chromosome fragment used to generate pJS091
prJS1342	AGGTTGCCGCTGGCCACGGCCG	Introducing D1104A mutation to *chvB* gene
prJS1343	TCGGCCGTGGCCAGCGGCAACCTC	Introducing D1104A mutation to *chvB* gene
prJS1402	CGCGCTTGGCCTCCCAGCCCATCC	Introducing R586A mutation to *chvB* gene
prJS1403	GCTGGGAGGCCAAGCGCGGCAAGTTGC	Introducing R586A mutation to *chvB* gene
prJS1404	TGCCGCGCGCGCGCTCCCAGCC	Introducing K587A mutation to *chvB* gene
prJS1405	GGGAGCGCGCGCGCGGCAAGTTGC	Introducing K587A mutation to *chvB* gene
prJS1406	CGCGCCCAGGCATGCTGGCGCGAGAC	Introducing R773A mutation to *chvB* gene
prJS1407	CCAGCATGCCTGGGCGCGTGGTG	Introducing R773A mutation to *chvB* gene
prJS1410	AGTCACCAGCCGCCCAGCGATGC	Introducing R776A mutation to *chvB* gene
prJS1411	GCTGGGCGGCTGGTGACTGGCAG	Introducing R776A mutation to *chvB* gene
prJS1700	CGATGTCATGATCTTTATAATCACCGTCATGGTCTTTGTA<br>GTCCATACCGGCACCCGTCGAGGTGAG	Generation of the Cgs 1-1540 truncation mutant
prJS1746	CGATGTCATGATCTTTATAATCACCGTCATGGTCTTTGTA<br>GTCGCCCACATGGCGCATCTGCACC	Generation of the Cgs 1-1846 truncation mutant
prJS1882	TATCGGCAGCAAGGGTCATGACATATTTGAC	Introducing D624A mutation to *chvB* gene
prJS1883	TGACCCTTGCTGCCGATACGCGCCTG	Introducing D624A mutation to *chvB* gene
prJS1884	CGCCAGGCGCTGACGAGCGAAAAATAGATC	Introducing D527A mutation to *chvB* gene
prJS1885	GCTCGTCAGCGCCTGGCGCGATGC	Introducing D527A mutation to *chvB* gene
prJS1886	CCACGGCCGCAACATAACGCGGTCCAAGCG	Introducing S1101A mutation to *chvB* gene
prJS1887	GTTATGTTGCGGCCGTGGACAGCGGC	Introducing S1101A mutation to *chvB* gene

Supplementary Table 3 | Plasmids generated for this study

Name	Plasmid background	Description
pJS091	pNPTD138	Introduces D624A/D626A/D739A mutations to the genomic *chvB* gene
pJS301	pNPTD138	Full length deletion of *chvB* gene
pJS305	pNPTD138	Adds a genomic C-terminal 3xFlag-tag to *chvB* gene
pJS309	pNPTD138	Introducing D2393A/D2528A mutations to the genomic *chvB* gene
pJS800	pBBR-MCS1	Empty control vector
pJS801	pBBR-MCS1	Rescue vector – cgs WT
pJS802	pBBR-MCS1	Rescue vector – cgs Y694A
pJS820	pBBR-MCS1	Rescue vector – cgs R586A
pJS821	pBBR-MCS1	Rescue vector – cgs K587A
pJS822	pBBR-MCS1	Rescue vector – cgs R773A
pJS823	pBBR-MCS1	Rescue vector – cgs R776A
pJS862	pBBR-MCS1	Rescue vector – cgs 1-1540 truncation mutant
pJS865	pBBR-MCS1	Rescue vector – cgs 1-1846 truncation mutant
pJS875	pBBR-MCS1	Rescue vector – cgs D527A
pJS876	pBBR-MCS1	Rescue vector – cgs D624A
pJS877	pBBR-MCS1	Rescue vector – cgs D739A
pJS878	pBBR-MCS1	Rescue vector – cgs D1104A
pJS879	pBBR-MCS1	Rescue vector – cgs E1300A
pJS880	pBBR-MCS1	Rescue vector – cgs D2528A
pJS881	pBBR-MCS1	Rescue vector – cgs S1101A

Supplementary Table 4 | *Agrobacterium* strains used in this study

Name	Carried plasmid	Description
Atu009	-	*A. tumefaciens* C58 with 3xFlag-tag insertion before the stop codon of *chvB*
Atu004	-	*A. tumefaciens* C58 with full length deletion of *chvB* gene
Atu125	-	Atu009 with additional D2393A/D2528A mutations in *chvB*
Atu130	-	Atu009 with additional D624A/D626A/D739A mutations in *chvB*
Atu243	pJS800	Atu004 with empty control vector
Atu219	pJS801	Atu004 with rescue plasmid encoding for Cgs WT
Atu220	pJS802	Atu004 with rescue plasmid encoding for Cgs Y694A
Atu230	pJS820	Atu004 with rescue plasmid encoding for Cgs R586A
Atu231	pJS821	Atu004 with rescue plasmid encoding for Cgs K587A
Atu232	pJS822	Atu004 with rescue plasmid encoding for Cgs R773A
Atu233	pJS823	Atu004 with rescue plasmid encoding for Cgs R776A
Atu239	pJS829	Atu004 with rescue plasmid encoding for Cgs D895C S919C
Atu260	pJS862	Atu004 with rescue plasmid encoding for Cgs 1-1540 truncation mutant
Atu355	pJS865	Atu004 with rescue plasmid encoding for Cgs 1-1846 truncation mutant
Atu362	pJS875	Atu004 with rescue plasmid encoding for Cgs D527A
Atu363	pJS876	Atu004 with rescue plasmid encoding for Cgs D624A
Atu364	pJS877	Atu004 with rescue plasmid encoding for Cgs D739A
Atu365	pJS878	Atu004 with rescue plasmid encoding for Cgs D1104A
Atu366	pJS879	Atu004 with rescue plasmid encoding for Cgs E1300A
Atu367	pJS880	Atu004 with rescue plasmid encoding for Cgs D2528A
Atu368	pJS881	Atu004 with rescue plasmid encoding for Cgs S1101A
