# Supplementary material for: Structure-function analysis of the cyclic β-1,2-glucan synthase from Agrobacterium tumefaciens
Source: Nat Commun. 2024 Feb 28;15:1844. doi: 10.1038/s41467-024-45415-8 (PMC10901819; doi:10.1038/s41467-024-45415-8)
Supplement: Supplementary file 3 — Reporting Summary [file 41467_2024_45415_MOESM3_ESM.pdf]

nature portfolio

Corresponding author(s): Christoph Dehio

Last updated by author(s): Jan 16, 2024

Reporting Summary

Nature Portfolio wishes to improve the reproducibility of the work that we publish. This form provides structure for consistency and transparency in reporting. For further information on Nature Portfolio policies, see our Editorial Policies and the Editorial Policy Checklist.

Statistics

For all statistical analyses, confirm that the following items are present in the figure legend, table legend, main text, or Methods section.

n/a	Confirmed	
☐	☒	The exact sample size (*n*) for each experimental group/condition, given as a discrete number and unit of measurement
☐	☒	A statement on whether measurements were taken from distinct samples or whether the same sample was measured repeatedly
☒	☐	The statistical test(s) used AND whether they are one- or two-sided<br>*Only common tests should be described solely by name; describe more complex techniques in the Methods section.*
☒	☐	A description of all covariates tested
☒	☐	A description of any assumptions or corrections, such as tests of normality and adjustment for multiple comparisons
☐	☒	A full description of the statistical parameters including central tendency (e.g. means) or other basic estimates (e.g. regression coefficient) AND variation (e.g. standard deviation) or associated estimates of uncertainty (e.g. confidence intervals)
☒	☐	For null hypothesis testing, the test statistic (e.g. *F*, *t*, *r*) with confidence intervals, effect sizes, degrees of freedom and *P* value noted<br>*Give P values as exact values whenever suitable.*
☒	☐	For Bayesian analysis, information on the choice of priors and Markov chain Monte Carlo settings
☒	☐	For hierarchical and complex designs, identification of the appropriate level for tests and full reporting of outcomes
☒	☐	Estimates of effect sizes (e.g. Cohen's *d*, Pearson's *r*), indicating how they were calculated

Our web collection on statistics for biologists contains articles on many of the points above.

Software and code

Policy information about availability of computer code

Data collection	SerialEM 3; MotionCor2; Focus 11; Digital Micrograph 3; EPU; No custom code was developed for this study
Data analysis	CryoSparc 2; CTFFIND4; UCSF Chimera 1.16; UCSF ChimeraX 1.7; Coot 0.9.4; Phenix 1.18; Molprobity; Origin 9.7; GraphPad Prism 9; Geneious Prime 2023; No custom code was developed for this study

For manuscripts utilizing custom algorithms or software that are central to the research but not yet described in published literature, software must be made available to editors and reviewers. We strongly encourage code deposition in a community repository (e.g. GitHub). See the Nature Portfolio guidelines for submitting code & software for further information.

Data

Policy information about availability of data

All manuscripts must include a data availability statement. This statement should provide the following information, where applicable:

- Accession codes, unique identifiers, or web links for publicly available datasets
- A description of any restrictions on data availability
- For clinical datasets or third party data, please ensure that the statement adheres to our policy

All data needed to evaluate the conclusions in the paper are present in the paper and/or the Supplementary Materials. PDB models generated in this study have been deposited at the Protein Data Bank with accession numbers of 8RF0 (CgsWT), 8RF9 (CgsiGP1), 8RFE (CgsiGP2), 8RFG (CgsiGP3). Cryo-EM maps determined in this study have been deposited at the Electron Microscopy Data Bank with accession numbers of EMD-19114 (CgsWT), EMD-19116 (CgsiGP1), EMD-19118 (CgsiGH2), EMD-19119 (CgsiGP3), EMD-19123 (CgsiGT). Raw data files and FragPipe output files related to the mass spectrometry analysis of Cgs glycosylation have

been deposited at MASSIVE and are accessible via the identifier MSV000093457 (https://massive.ucsd.edu/ProteoSAFe/dataset.jsp?task=597a8c43a0634bc59c84a800d80adb6d)

Research involving human participants, their data, or biological material

Policy information about studies with human participants or human data. See also policy information about sex, gender (identity/presentation), and sexual orientation and race, ethnicity and racism.

Reporting on sex and gender	n/a
Reporting on race, ethnicity, or other socially relevant groupings	n/a
Population characteristics	n/a
Recruitment	n/a
Ethics oversight	n/a

Note that full information on the approval of the study protocol must also be provided in the manuscript.

Field-specific reporting

Please select the one below that is the best fit for your research. If you are not sure, read the appropriate sections before making your selection.

- [x] Life sciences
- [ ] Behavioural & social sciences
- [ ] Ecological, evolutionary & environmental sciences

For a reference copy of the document with all sections, see nature.com/documents/nr-reporting-summary-flat.pdf

Life sciences study design

All studies must disclose on these points even when the disclosure is negative.

Sample size	No statistical methods were used to pre-determine sample sizes. Our sample sizes are similar to those reported in previous publications (Sedzicki et al., NSMB 2022).
Data exclusions	No data was excluded
Replication	All growth curves represent 3 biological replicates (n=3) in technical duplicates (2 wells of a 96-well plate per strain tested);<br>In vitro glucan synthesis assay: representative result of two measurements (n=2)<br>All attempts at replication were successful.
Randomization	No randomization was performed. Experimental groups were treated identical, thus randomization is not required.
Blinding	Blinding was not necessary. All measurements were quantified by automated machines, and no data were excluded.

Reporting for specific materials, systems and methods

We require information from authors about some types of materials, experimental systems and methods used in many studies. Here, indicate whether each material, system or method listed is relevant to your study. If you are not sure if a list item applies to your research, read the appropriate section before selecting a response.

Materials & experimental systems

n/a	Involved in the study	
[ ]	[x]	Antibodies
[x]	[ ]	Eukaryotic cell lines
[x]	[ ]	Palaeontology and archaeology
[x]	[ ]	Animals and other organisms
[x]	[ ]	Clinical data
[x]	[ ]	Dual use research of concern
[x]	[ ]	Plants

Methods

n/a	Involved in the study	
[x]	[ ]	ChIP-seq
[x]	[ ]	Flow cytometry
[x]	[ ]	MRI-based neuroimaging

Antibodies

Antibodies used	Anti-Flag antibody clone M2, Sigma-Aldrich F1804; Anti-mouse IgG, HRP-linked Antibody #7076

Validation	https://www.sigmaaldrich.com/specification-sheets/469/360/F1804-5MG_________SIGMA____.pdf<br>https://www.cellsignal.com/product/productDetail.jsp?productId=7076&country=CA

Plants

Seed stocks	*Report on the source of all seed stocks or other plant material used. If applicable, state the seed stock centre and catalogue number. If plant specimens were collected from the field, describe the collection location, date and sampling procedures.*
Novel plant genotypes	*Describe the methods by which all novel plant genotypes were produced. This includes those generated by transgenic approaches, gene editing, chemical/radiation-based mutagenesis and hybridization. For transgenic lines, describe the transformation method, the number of independent lines analyzed and the generation upon which experiments were performed. For gene-edited lines, describe the editor used, the endogenous sequence targeted for editing, the targeting guide RNA sequence (if applicable) and how the editor was applied.*
Authentication	*Describe any authentication procedures for each seed stock used or novel genotype generated. Describe any experiments used to assess the effect of a mutation and, where applicable, how potential secondary effects (e.g. second site T-DNA insertions, mosiacism, off-target gene editing) were examined.*
